# Quaternary to recent uplift rates of salt diapirs in the Romanian Carpathians determined from radiocarbon dating and PSInSAR data

**DOI:** 10.1038/s41598-025-08293-8

**Published:** 2025-07-02

**Authors:** Dan Mircea Tamas, Alexandra Tămaș, Irka Hajdas, Gabriela Odilia Sava, Valentin Poncos, Delia Teleaga

**Affiliations:** 1 Department of Geology and Center for Integrated Geological Studies, Babeș-Bolyai University, Cluj-Napoca, Romania; 2 Laboratory for Ion Beam Physics, Swiss Federal Institute of Technology (ETH), Zurich, Switzerland; 3 Horia Hulubei National Institute for R&D in Physics and Nuclear Engineering, Magurele, Romania; 4 Terrasigna, Bucharest, Romania

**Keywords:** Tectonics, Structural geology, Geomorphology

## Abstract

Understanding the past and present-day dynamics of salt structures is critical due to their scientific significance and societal and economic implications. Past and present salt movement can trigger hazards like sinkholes and landslides, posing risks to inhabited areas and industries such as mining, hydrocarbon extraction, and energy storage. Moreover, there is a considerable data gap regarding uplift rates of salt diapirs in humid continental climates. In this study, we investigate the relative uplift rates of the Manzalesti salt diapir, one of Europe’s largest rocksalt outcrops. We combine radiocarbon dating of vegetal fragments in sediment-filled dissolution notches (paleo-river terraces) and detailed vertical offsets extracted from digital outcrop models to calculate relative rock uplift rates. The results reveal relative salt uplift rates of 10.5 ± 0.8 and 29.8 ± 4.2 mm/year over the past ~ 720 years. We also compared these results against recent PSInSAR-derived velocities that confirm average relative vertical rates of 10 mm/year, peaking at 55 mm/year, filling a key data gap for uplift rates of salt diapirs in humid continental climates.

**Supplementary Information:**

The online version contains supplementary material available at 10.1038/s41598-025-08293-8.

## Introduction

Salt has fascinated and aided the development of humanity for millennia worldwide, including in the Romanian Carpathians (e.g^1^). It is also among the solutions for the energy transition from a mainly fossil-fuel society to more sustainable and environmentally friendly energy resources (e.g. hydrogen storage^2,3^;).

Because of its physical and mechanical properties, salt can be considered a geological fluid^4,5^. Under both high and low confining pressures, salt often rises to the surface in the form of salt diapirs^5^ or creates different kinds and shapes of salt structures.

Quantifying the rates of salt flow and diapir rise in the geological past, but also in the historical past and present-day, has always been of importance when it comes to understanding the deformation of salt. The recent (e.g. Quaternary) to present-day deformation rates can be especially important for areas where localities are built on top of salt structures, for hydrocarbon fields located near-, supra- or sub-salt, or for industry projects related to storage of hydrogen in artificial salt caverns.

Several methods have been employed to understand the timing and rates of salt rise, salt flow and salt-sediment interaction^5^such as seismic interpretation (e.g^6^)., analogue and numerical modelling (e.g^7–9^)., field-based interpretations (e.g^10,11^)., microstructural observations (e.g^12,13^). and remote sensing observations (e.g^14–18^). More precise dating of the Quaternary movements of salt structures has been achieved through the dating of marine terraces, dissolution grooves, and cave systems (e.g^19–24^).

**Fig. 1:**
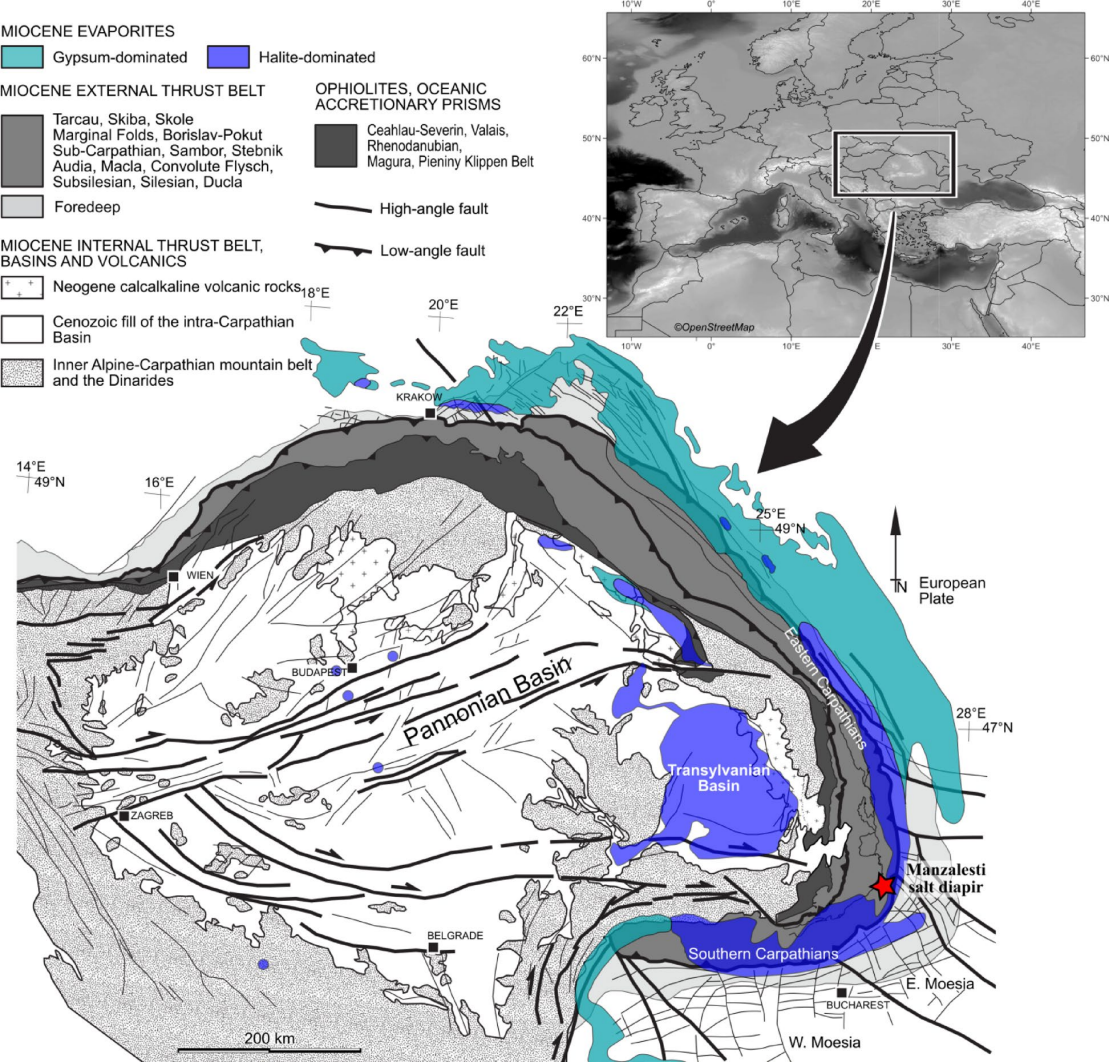
Map of the major Miocene structural lineaments and features of the Pannonian–Carpathian area showing the type and approximate present-day distribution of the Miocene evaporites (after^3,25–27^ and references therein). The location of the Manzalesti salt diapir is marked with a red star. The inset map was generated using QGIS v. 3.10.1 (https://qgis.org/) with map data retrieved from OpenStreetMap.

The Romanian Carpathians (Fig. 1) provide a natural laboratory for the study of salt, from multiple points of view, including archeology and anthropology^28^karstic features^29,30^salt tectonics^26,27,31^ and geoheritage^32^. The Carpathians (Fig. 1) are an Alpine orogen that records the closure of the Alpine Tethys^33^. The area of interest is of major importance to the study of salt tectonics as it provides unique surface exposures, salt mines and caves, and subsurface data like seismic and well data. The age of the most widespread evaporites in Romania is Miocene^3^; Fig. 1). These evaporites were involved in the main emplacement of the Carpathians (middle Miocene), providing detachment levels for major thrusts, as well as being reactivated during the upper Miocene to recent deformation event (known as the Wallachian phase^34^).

During Quaternary times, the Romanian Carpathians experienced strong exhumation, especially in the Carpathian Bend Zone, where the exhumation was calculated to be as high as 5 km^35^. It is during these recent deformation events that most of the diapirs that still outcrop today were emplaced^26^. Due to the fact that the studied area is in a humid continental climate with an average annual rainfall of ~ 500 mm^36^most of these salt outcrops experience significant karstification and dissolution^29–31^.

**Fig. 2:**
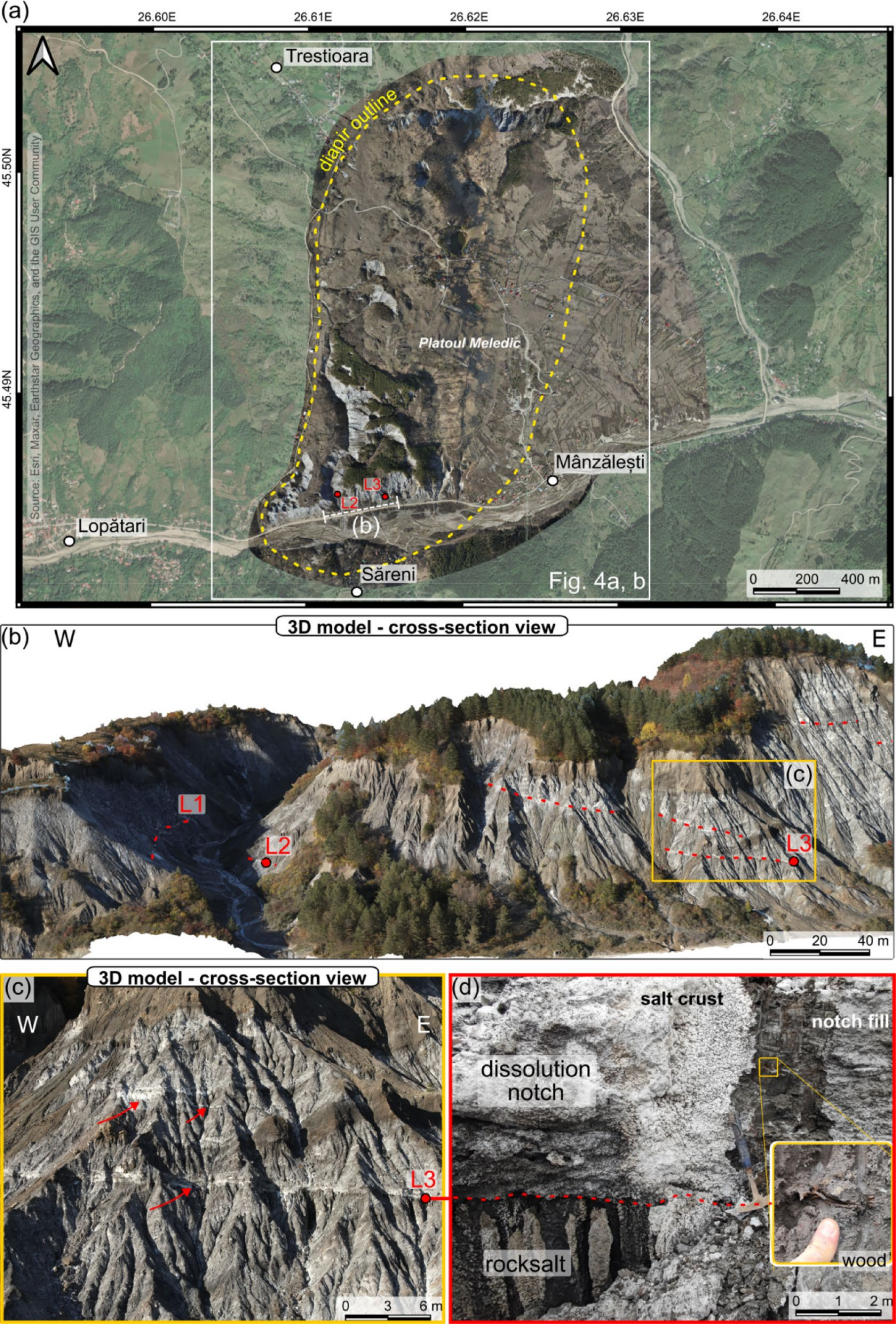
(**a**) Satellite map with detailed orthomosaic overlay of the area of interest, highlighting sample locations and diapir outline. The map was created in QGIS v. 3.10.1 (https://qgis.org/) and the source of the satellite base layer is Esri, Maxar, Earthstar Geographics, and the GIS User Community. (**b**) Digital outcrop model of the sampled area, showing dissolution notches (red dashed lines) and the sampled layers (numbered L1 to L3). (**c**) close-up aerial photograph illustrating the dissolution notches (marked with red arrows) and the location of layer 3. (**d**) photograph of layer 3 sample location and the white crust that is covering the dissolution notch. Note the wood fragment exposed along with the sediments (e.g. Figure 3).

Based on outcrop, microstructural, and UAS-based study of the Manzalesti salt diapir (Figs. 1 and 2a), Tămaș et al.^31^ identified near-horizontal white surfaces that were interpreted as dilatant shear zones within the salt. However, the authors highlighted the uncertainty of this interpretation as these surfaces could not be safely reached and sampled at the time. Since then, we have identified similar surfaces that were safely reached, sampled, and studied.

In this contribution, we not only provide a new interpretation for the origin of those near-horizontal white surfaces as sediment-filled dissolution notches but also date them using radiocarbon dating and calculate relative uplift rates of the Manzalesti salt diapir. We augment these findings with PSInSAR-derived vertical velocities to discuss the present-day movement of this salt structure. The implications of these findings and calculated rates are further discussed within the paper.

We hope that this work will encourage others to apply this approach, allowing us to better understand salt deformation in multiple environments and reduce the risks associated with the present-day movement of salt structures for applications such as drilling, storage in salt caverns, and hazards. We also hope that these sediment-filled dissolution notches will spark the interest of archaeologists as they might preserve artifacts from historical salt exploitations.

## Dissolution notches cross-cutting salt bedding

White-coloured, low-angled, and often cross-cutting planes were identified on the surface exposures of the Manzalesti salt diapir^31^. Initially interpreted as possible dilatant shear surfaces made within the halite, they are planar voids filled with sediments and covered by a thin layer of white recrystallized halite crust (Fig. 2d). This white crust is caused by the highly saturated brine flowing through the layer, followed by the evaporation of water at the surface of the outcrop (Fig. 2).

In the area of the Manzalesti salt diapir (Figs. 1 and 2), three such layers were safely reached, studied, and sampled. Upon detailed examination, the material within these layers is formed of unconsolidated clastic material. Two of the layers (layers 2 and 3; Fig. 2) also contain vegetal fragments (wood and leaves; Fig. 2d).

## Radiocarbon ages of vegetal fragments preserved in the dissolution notches and relative salt uplift rates

**Fig. 3:**
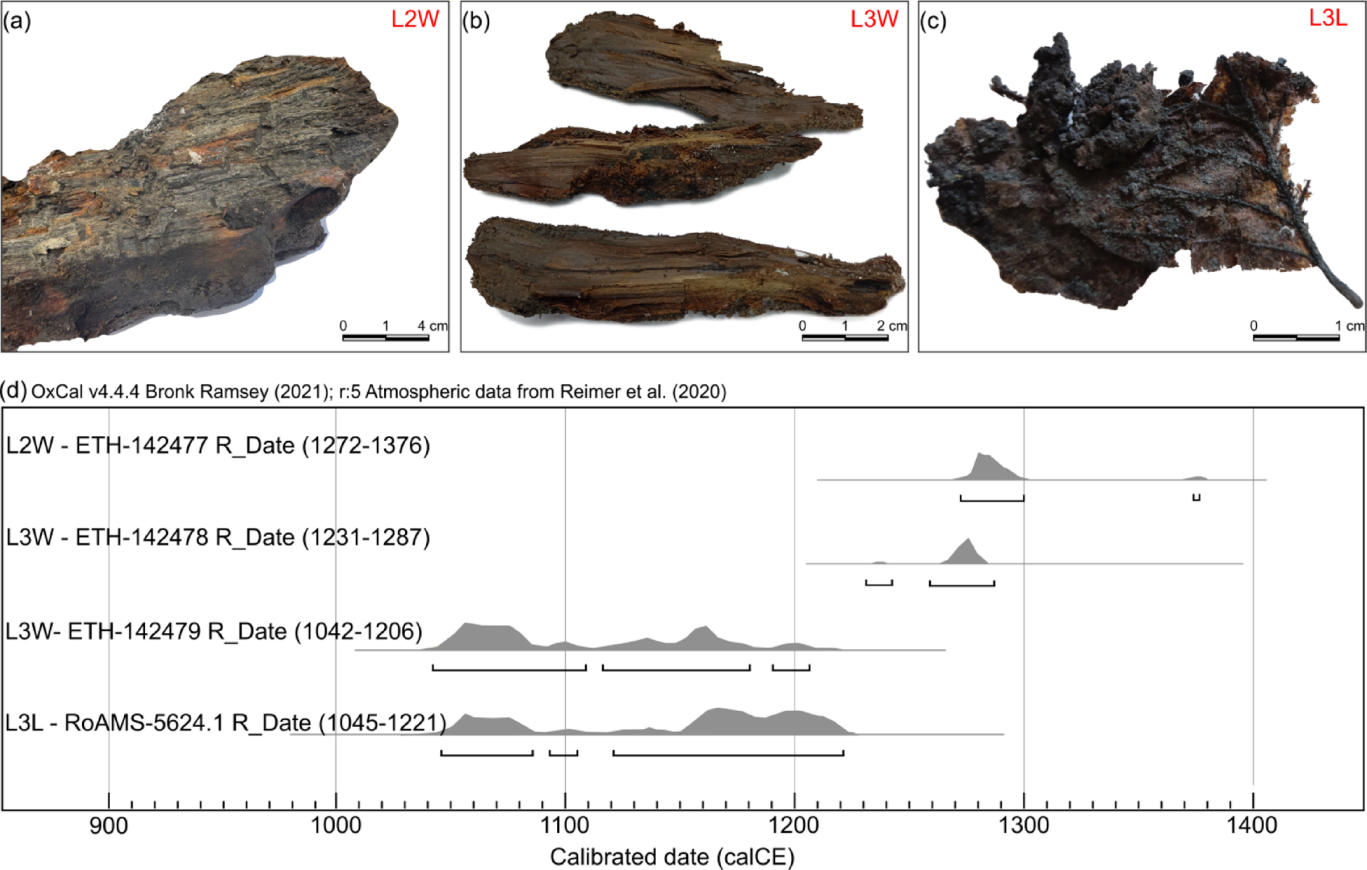
Dated vegetal material (**a**-**c**) and results of the calibration using OxCal 4.4^37^ and INTCAL2020^38^ (**d**). (**a**) photograph of the wood sample from layer 2. (**b**) photograph of the wood samples from layer 3. (**c**) photograph of the leaf sample from layer 3. The wide range of calibrated ages for the older samples (ETH-142479 and RoAMS-5624.1) in layer 3 is due to the variability of the calibration curve^39^ methods.

The identified vegetal material (Fig. 3a-c) was used for radiocarbon dating to better constrain the ages of the studied dissolution grooves. One wood fragment from layer 2 provided a^14^C age range of 1272–1376 calCE (Fig. 3a). The leaf from layer 3 (Fig. 3c) provided an age of 1045–1221 calCE, while the wood fragments from the same layer provided ages of 1231–1287 calCE and 1042–1206 calCE (Fig. 3b).

Detailed digital outcrop models of centimeter-level accuracy (e.g. Figure 2b) were created and used to calculate the precise vertical distance between dated dissolution notches (Fig. 2) and the water surface level of the stream and the main river. For each terrace, the elevation was measured at the sample location and compared to the present-day riverbed elevation at the nearest orthogonal point. In the case of the terrace intersected by the stream cutting through the diapir (layer 2; Fig. 2b), the comparison was made directly at the stream channel adjacent to the sample site. While the spatial accuracy of these measurements is high, we acknowledge that uncertainties exist, particularly concerning the long-term stability of the river base level. Therefore, the reported uplift rates should be understood as relative values, reflecting the current difference in elevation between the sampled terrace and the modern stream level.

For the purpose of estimating salt diapir uplift rates, we have chosen to use the radiocarbon age ranges for the youngest samples obtained from each terrace layer (Fig. 3). This approach is commonly used in biostratigraphic and geochronological studies, particularly in contexts where older material may be reworked or contain inbuilt age (e.g., wood from the inner core of trees). Such materials can yield significantly older dates that do not accurately reflect the timing of sediment deposition. In dynamic fluvial and hillslope environments, older plant material can be eroded, transported, and redeposited, leading to a mix of age signals within a single layer.

The measured elevation differences (7.3 m for layer 2 and 25.1 m for layer 3), along with the youngest age intervals of the studied layers (1272–1376 calCE for layer 2 and 1231–1287 calCE for layer 3; Fig. 3), provided the necessary data to calculate relative rock uplift rates. During the past ~ 720 years, the studied area of the Manzalesti salt diapir (southern area) experienced relative uplift rates of 10.5 ± 0.8 mm/year for layer 2 and 32.8 ± 1.2 mm/year for layer 3. If we use the entire age interval for layer 3 (1042–1287 calCE), we obtain an average relative uplift rate of 29.8 ± 4.2 mm/year.

## Present-day vertical velocities based on PSInSAR

**Fig. 4:**
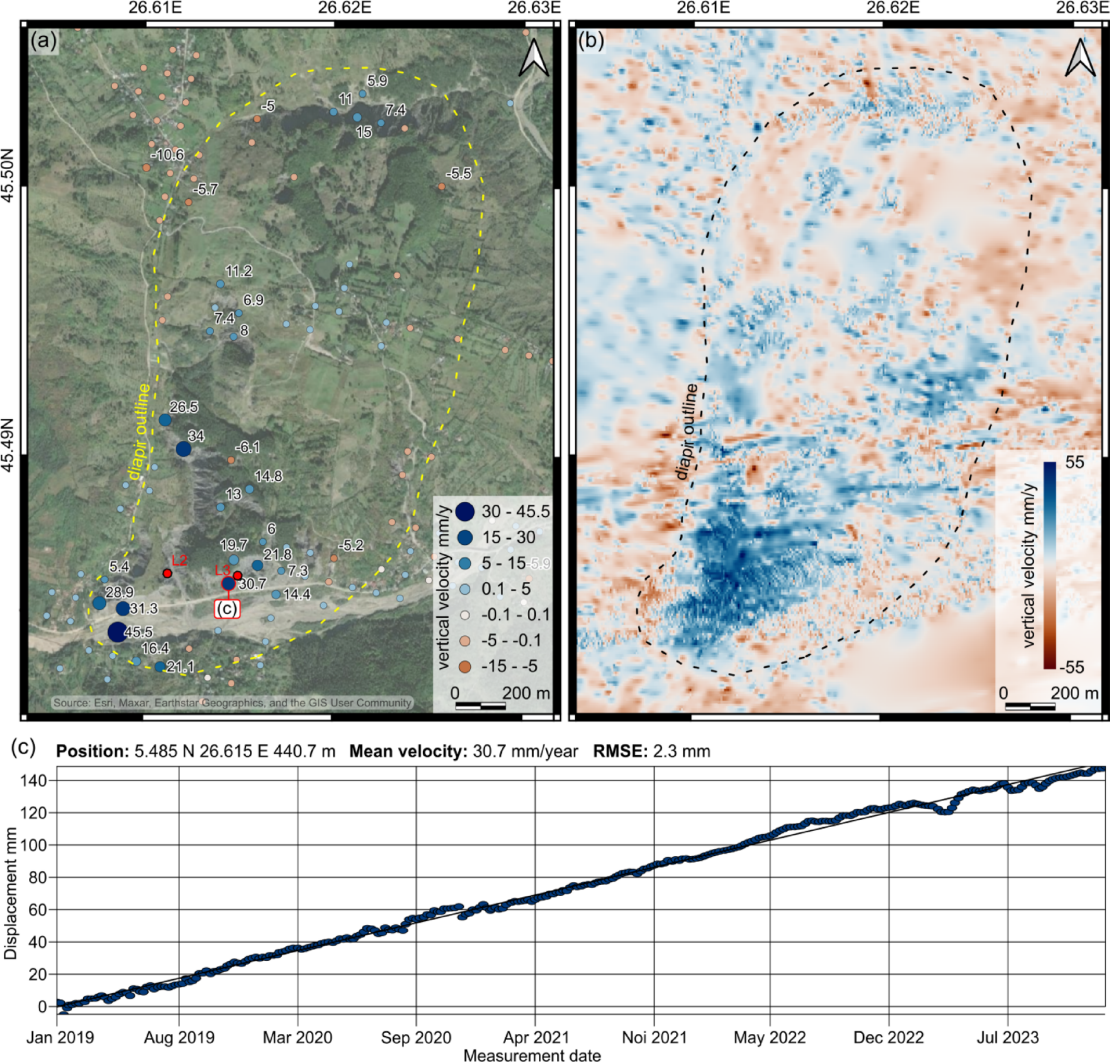
PSInSAR results illustrating the vertical velocities in the area of the Manzalesti salt diapir. (**a**) Figure showing the average vertical velocities between 2019 and 2023, using data retrieved from the European Ground Motion Service^40,41^. The map was created in QGIS v. 3.10.1 (https://qgis.org/) and the source of the satellite data is Esri, Maxar, Earthstar Geographics, and the GIS User Community. (**b**) Map showing the distribution of average vertical velocities obtained by Terrasigna using the Sentinel-1 data (2018–2020). Note the high vertical displacements in the south and southwestern areas of the salt diapir. (**c**) Vertical displacement profile of the point closest to sample location of Layer 3. The data was retrieved from the European Ground Motion Service^40,41^.

The openly available PSInSAR data from the European Ground Motion Service (EGMS^40,41^;) was used to understand the recent (2019–2023) rates and distribution of vertical movement in the area of the Manzalesti salt diapir (Fig. 4a, c). The studied area exhibits average vertical velocities above the salt diapir area of 10 mm/year and are as high as 45.5 mm/year in the southwestern part of the diapir (Fig. 4a).

By reprocessing the Sentinel-1 data (Fig. 4b – April 2018 to September 2020), we significantly improved the mapping of the vertical dynamics of the salt diapir, allowing for a more detailed assessment of its movement (original pixel size of ~ 24 m²). We extracted the vertical component of motion and generated a high-resolution vertical motion map (Fig. 4b), revealing that while the area surrounding the salt diapir and in the northern part of the diapir exhibits either slow subsidence or negligible movement, extensive areas of the salt diapir are experiencing rock uplift. Notably, the highest vertical velocities (peaking at 55 mm/year) are concentrated in the southwestern part of the diapir. The average vertical velocity estimated derived from radiocarbon dating for the past ~ 720 years are in good alignment with the PSInSAR results. While the results presented in Fig. 4a and b are based on slightly different time intervals, their overlapping spatial and temporal coverage helps validate the observed deformation trends.

## The nature and implications of dissolution notches

**Fig. 5:**
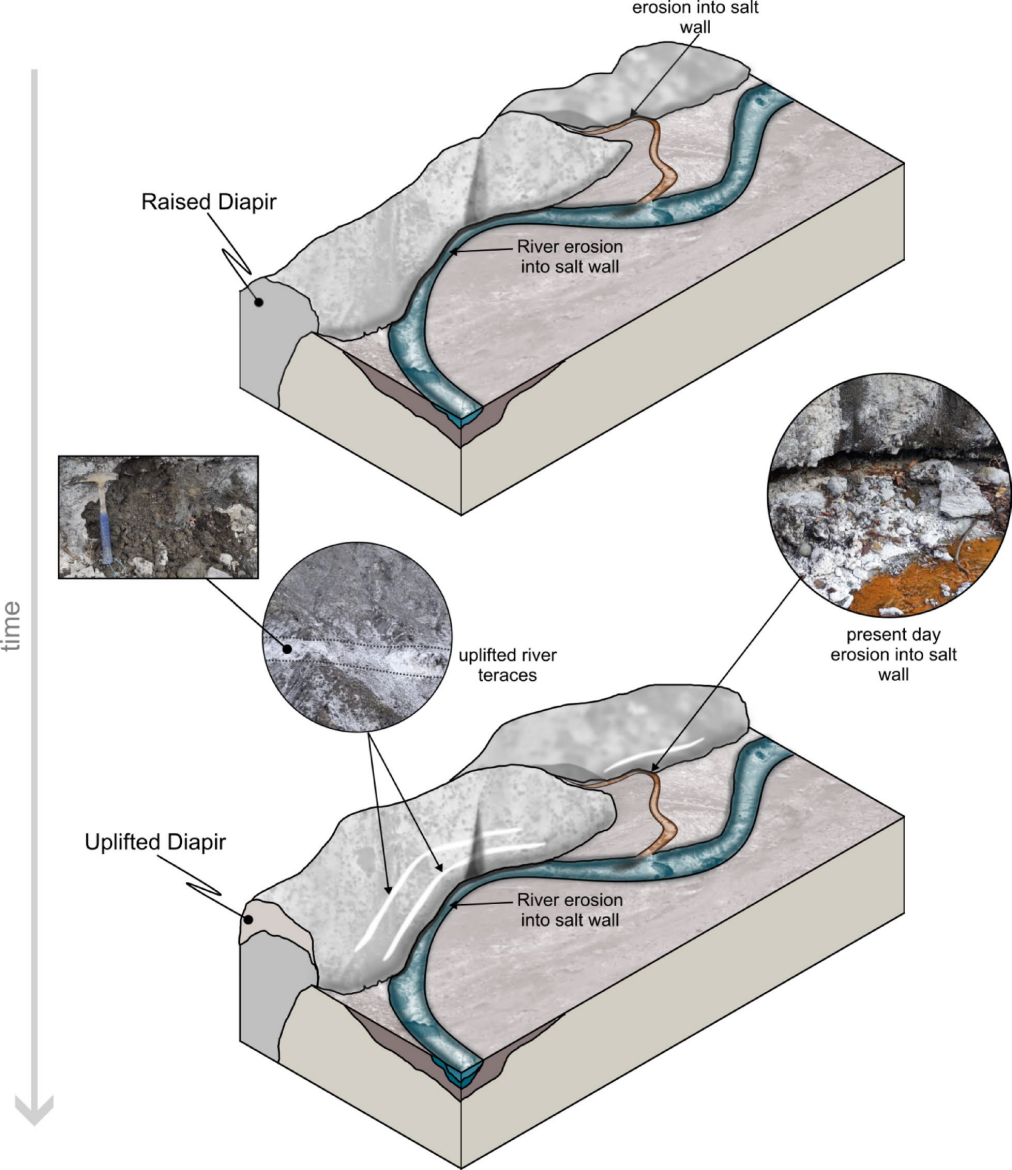
Block model depicting the evolution of dissolution notched during salt diapir uplift. Note the present-day erosion into the salt wall with the generation of such dissolution notches and fill with similar clastic material.

We now interpret the sediment-filled planar surfaces (Fig. 2a-c) as being dissolution notches created by paleo-river terraces, which were dissolved by the streams and rivers crossing the diapir. The sediments deposited in these notches (Fig. 2c) were exhumed as the diapir was rising and are now a testament to relative uplift rates (Fig. 5). There are alternative possible explanations for the presence and timing of emplacement of the plant material. Colluvial or hillslope processes could possibly bring material and unconsolidated debris on the slope of the salt wall, as often seen within the valleys. Bioturbation could also represent a possible source. To mitigate these possible scenarios, the samples have been taken from walls that have been clearly exposed to weathering and dissolution, and not from the near surface, but ~ 20 cm of the material in the layer was removed before sampling. One other confirmation of the mechanism of formation of these dissolution notches is that nowadays, one can observe the same erosional/dissolution process, with the deposition of similar sediments at the base of the salt outcrop, in contact with the streams crossing this outcrop (Figs. 2 and 5).

Similar white layers, as the ones in this study, have been observed by the authors in other salt outcrops along the Romanian Carpathians (e.g. Coza, Sarea Rosie, Slanic Prahova) and in the Transylvanian Basin (Praid, Sovata) as well as mentioned in the literature (e.g. Slanic Prahova and Praid)^42,43^. If technically possible (e.g. presence of plant material), the dating of these surfaces can provide critical insights into the evolution and surface dynamics of salt structures in the Romanian Carpathians and Transylvanian Basin.

The relative vertical velocities of 10.5 ± 0.8 and 29.8 ± 4.2 mm/year calculated for the southern rim of the Manzalesti salt diapir based on the vertical difference between the present-day rivers and the river terraces/dissolution grooves (formed ~ 720 years ago), as well as the PSInSAR derived values (peaking at 55 mm/year) fit well within the values compiled by Jackson and Hudec^5^ from salt diapirs in Iran, Yemen and Israel (1–80 mm/year). Some of the higher vertical velocity values provided by Talbot et al.^44^ for the Kuh-e-Jahani diapir (up to 3000 mm/y) have recently been disputed^24^.

The measured recent rise of the Manzalesti salt diapir is interpreted to be driven by a combination of gravitational and displacement loading, specifically, by the weight of the rocks above the salt layer and the shortening of the diapir stem due to ongoing compression^31^. Furthermore, our results show that^14^C dating of sediments in dissolution notches created by paleo-rivers represents a successful method through which the Quaternary uplift rates of salt diapirs can be calculated. The rates calculated using the^14^C ages are interpreted as relative values, influenced by both the complex geometry of the terraces and the dynamic behavior of the salt body. Many of the terraces are not parallel to one another or to the modern river channel, which likely reflects post-depositional tilting and deformation associated with salt movement. This complexity, combined with the unknown incision history of the river, introduces uncertainty in distinguishing between fluvial downcutting and true salt rise. The values obtained from the valleys cutting the salt structure are treated as less certain in comparison to the values obtained from the edge of the salt diapir, where the main river resides. Additionally, in areas where the salt body spreads laterally (as illustrated in Fig. 5), the apparent surface uplift may not directly correspond to vertical displacement of the diapir core. Instead, these measurements capture a combination of vertical and lateral salt flow, particularly at the flanks. Combined with PSInSAR, these recent^14^C results offer important insights into the dynamics of the Manzalesti salt diapir, showing that at least for the past ~ 720 years, this structure has been rising with average vertical velocities of up to ~ 10 mm/year (average vertical velocity calculated from all EGMS data points within the defined area of interest for the 2019–2023 time interval).

If we use the average vertical velocities (~ 10 mm/year) and the highest point of this salt structure above the regional (~ 170 m) and extrapolate these values back in time, it means that the present-day top of the Manzalesti salt diapir could have been at regional height ~ 17,000 years ago. Hence, it is even possible that what is today regarded as one of the largest halite outcrops in Europe might not even have been exposed to the surface c. 17,000 years ago. This young age could also explain why this outcrop is still preserved in a climate with an average annual rainfall of ~ 500 mm/year, prone to dissolving halite at high rates.

## Methods

### Radiocarbon dating

At the ETH laboratory, the wood samples were treated with Acid - Base_- Acid to remove contamination^45^. The first step of acid wash (0.5 M HCl, 60 °C) was extended to 25 h. It was followed by base (0.1 M NaOH, 60°C_ and 2nd acid wash (0.5 M HCl, 60 °C), for 2 h each. The dry, clean wood (ca. 3.5 mg), was transferred to Al cups for combustion in Elemental Analyzer and subsequent graphitization^46^. Graphite was pressed into Al cathodes and analyzed using the AMS system LEA^47^. The measured ^14^C/^12^C ratio of wood was corrected for blank values and normalized to a standard HOx2^48^. Calibration was performed using OxCal 4.4^37^ and INTCAL20 calibration curve^38^.

At RoAMS laboratory the leaf sample was treated with Acid-Base-Acid identic with the protocol available for vegetal remains described in Sava et al.^49^. The cleaned sample was then combusted and graphitized using an Elemental Analyzer coupled with an AGE3 system^50^. The sample was analyzed on a High Voltage Engineering AMS system^49^corrected for blank and ^13^C/^12^C fractionation and subsequently normalized to HOx2^48^ standard. Calibration was performed using OxCal 4.4^37^ and INTCAL20 calibration curve^38^.

### Photogrammetry

Photogrammetry was used to create a high-resolution digital outcrop model of the sample locations and to measure the precise height difference between the sample sites and the present-day level of the river and streams. This approach enabled a detailed visual assessment of the spatial distribution of geological structures across multiple scales (centimeters to meters) and provided access to otherwise inaccessible outcrops in steep or high-relief settings. The models were based on both newly acquired UAV photogrammetry data and the datasets of Tămaș et al.^31^. For the newly acquired data, images were collected using a DJI Matrice 300 RTK uncrewed aerial vehicle (UAV) equipped with a DJI Zenmuse P1 photogrammetry camera (45 MP). The UAV surveys employed both manual and automatic photograph acquisitions to optimize image coverage and ensure a high degree of overlap between images.

The creation of the 3D digital outcrop models was performed using Agisoft Metashape Professional and DJI Terra. More details on the methodology are available in Tămaș et al.^31^and the models are provided as supplementary data to that study. The final models were used for visualization and vertical offset measurements.

### PSInSAR

In this study, we employed Persistent Scatter Interferometric Synthetic Aperture Radar (PSInSAR) as our primary method for measuring present-day vertical ground motion velocities, using Sentinel-1 SAR data processed by the European Ground Motion Service (2019–2023) and by Terrasigna (2018–2020). The European Ground Motion Service EGMS, an integral part of the Copernicus Land Monitoring Service, enables the high-resolution monitoring of ground deformations across participating European countries^40,41^. This service uses advanced persistent scatterer and distributed scatterer PSInSAR processing techniques, leveraging the full-resolution acquisition of Sentinel-1 radar imagery (20 × 5 m). It provides millimeter/year-scale accuracy for detecting and measuring deformations associated with landslides, subsidence, and infrastructure stability^40,41^. For our analysis, we accessed the EGMS “Ortho” product, specifically the vertical movements between 2019 and 2023^41^ at a spatial resolution of 10,000 m^2^. The data is distributed as a vector map of measurement points, each featuring an annotated geolocation, quality measures, and corresponding time series of displacement^40,41^. The datasets can be easily visualized and downloaded from https://egms.land.copernicus.eu/.

The EGMS data provides reliable yet localized information regarding the average vertical velocities. As our goal is to better understand the distribution of vertical motion and diapir dynamics over its entire area, we used the same Sentinel-1 data (collected between April 2018 and September 2020) and reprocessed it at Terrasigna. The in-house PSInSAR algorithms (as described by^51^ are similar to the ones employed by EGMS but focused on local processing. Local processing has the advantage of choosing a more suitable reference point near the area of interest and of detecting the maximum number of radar targets on the ground by refining processing parameters and doing a careful quality analysis. By maximizing the spatial density of the measurement points on the ground and avoiding spatial interpolation, the resulting map (Fig. 4b) is more accurate than a map obtained from interpolating a low number of measurement points (as it would be required fom EGMS data). By reprocessing the Sentinel-1 data, 14323 points (Path 109 Descending - all scenes between April 1, 2018 and September 29, 2020) and 179692 points (Path 131 Ascending - all scenes between April 8, 2018 and Sept 30, 2020) were detected and measured in slant-range direction (radar line of sight). We used this data to extract the vertical component of the motion by combining ascending and descending measurements at the original pixel size (roughly 24 m^2^) and to generate the vertical motion map illustrated in Fig. 4b, as explained below.

For the purposes of 3D decomposition, data multi-looking (averaging and downsampling) was applied with a 5 × 1 window, producing aggregated pixels of approximately 10 × 12 m². This step was essential to align radar targets imaged from ascending and descending satellite orbits, which do not perfectly overlap due to geometric differences. Only those multi-looked pixels that contained at least one valid radar target from each orbit contributed to the final 3D velocity map. The process inherently reduces spatial resolution from roughly 24 m^2^ per pixel to 120 m^2^ per pixel.

While PSInSAR is a powerful technique for measuring ground motion, it comes with several limitations and uncertainties. Displacement measurements can only be obtained from areas where the radar signal is reflected back to the sensor, meaning that bodies of water and dense vegetation typically scatter the signal away and dynamics cannot be measured. Furthermore, scattering areas must remain unchanged over the entire observation period, often spanning years, so that their radar signatures can be consistently identified. Even minor changes to the surface, such as a vehicle driving across it, can alter the radar signature and introduce incoherence.

Measuring ground motion without installing physical sensors on the ground is inherently complex, as multiple overlapping mechanisms contribute to surface movement. In this case, a strong overall rising trend is apparent, but variations in motion direction and amplitude suggest the presence of localized subsidence from sinkholes or slope-related dynamics. To capture the full spatial resolution of the velocity field, all observed dynamics were retained, acknowledging the trade-off that some low-amplitude, unrelated motion might be included. Nonetheless, the sparse EGMS data (Fig. 4a) serves as a useful validation for the more spatially dense measurements (Fig. 4b).

For the visual representation of the PSInSAR results (Fig. 4a, b), we used the scientific color map vik^52^ to ensure an accurate depiction of the data while preventing perceptual distortion and exclusion of readers with color-vision deficiencies^53^.

## Electronic supplementary material

Below is the link to the electronic supplementary material.

Supplementary Material 1

Supplementary Material 2

## Data Availability

The results of the radiocarbon dating of the four samples presented in this manuscript are provided as supplementary material. The digital outcrop models used for calculating the vertical offset between the river beds and the sampled layers are taken from Tamas et al. 2021 and are available at https://doi.org/10.5281/zenodo.4889107.

## References

[1] Brigand, R. & Weller, O. Neo-Eneolithic settlement pattern and salt exploitation in Romanian Moldavia. *J. Archaeol. Science: Rep.* **17**, 68–78 (2018).

[2] Duffy, O. et al. The Role of Salt Tectonics in the Energy Transition: An Overview and Future Challenges. *tekt* 1, (2023).

[3] Tămas, D. M. et al. A review of salt tectonics in romania’s Transylvanian basin and implications for energy transition. *SP* **555**, (2025). SP555-2024-9. 10.1144/SP555-2024-9

[4] Warren, J. K. *Evaporites: A Geological Compendium* (Springer International Publishing, 2016). 10.1007/978-3-319-13512-0

[5] Jackson, M. P. A. & Hudec, M. R. *Salt Tectonics: Principles and Practice* (Cambridge University Press, 2017). 10.1017/9781139003988

[6] Rowan, M. G., Tilton, J., Lebit, H. & Fiduk, J. C. Thin-skinned extensional salt tectonics, counterregional faults, and the Albian gap of Brazil. *Mar. Pet. Geol.* **137**, 105478 (2022).

[7] Talbot, C. & Aftabi, P. Geology and models of salt extrusion at Qum kuh, central Iran. *JGS* **161**, 321–334 (2004).

[8] Peel, F. J., Hudec, M. R. & Weijermars, R. Salt diapir downbuilding: fast analytical models based on rates of salt supply and sedimentation. *J. Struct. Geol.* **141**, 104202 (2020).

[9] Adamuszek, M., Tămaş, D. M., Barabasch, J. & Urai, J. L. Rheological stratification in impure rock salt during long-term creep: morphology, microstructure, and numerical models of multilayer folds in the Ocnele Mari salt mine, Romania. *Solid Earth*. **12**, 2041–2065 (2021).

[10] Alsop, G. I., Weinberger, R., Levi, T. & Marco, S. Cycles of passive versus active diapirism recorded along an exposed salt wall. *J. Struct. Geol.* **84**, 47–67 (2016).

[11] Zucker, E., Frumkin, A., Agnon, A. & Weinberger, R. Internal deformation and uplift-rate of salt walls detected by a displaced dissolution surface, dead sea basin. *J. Struct. Geol.* **127**, 103870 (2019).

[12] Urai, J. L., Spiers, C. J., Zwart, H. J. & Lister, G. S. Weakening of rock salt by water during long-term creep. *Nature* **324**, 554–557 (1986). 29517720 10.1038/324554a0

[13] Schléder, Z., Urai, J. L., Nollet, S. & Hilgers, C. Solution-precipitation creep and fluid flow in halite: a case study of Zechstein (Z1) Rocksalt from Neuhof salt mine (Germany). *Int. J. Earth Sci. (Geol Rundsch)*. **97**, 1045–1056 (2008).

[14] Weinberger, R., Lyakhovsky, V., Baer, G. & Begin, Z. B. Mechanical modeling and InSAR measurements of Mount sedom uplift, dead sea basin: implications for effective viscosity of rock salt. *Geochem. Geophys. Geosyst.* **7**, 2005GC001185 (2006).

[15] Weinberger, R. et al. Quaternary rise of the sedom diapir, dead sea basin. in New Frontiers in Dead Sea Paleoenvironmental Research (Geological Society of America, doi:10.1130/2006.2401(03). (2006).

[16] Aftabi, P., Roustaie, M., Alsop, G. I. & Talbot, C. J. InSAR mapping and modelling of an active Iranian salt extrusion. *J. Geol. Soc.* **167**, 155–170 (2010).

[17] Barnhart, W. D. & Lohman, R. B. Regional trends in active diapirism revealed by mountain range-scale InSAR time series: ACTIVE ZAGROS DIAPIRISM. *Geophys. Res. Lett.* 39, n/a-n/a (2012).

[18] Manea, V. C., Armaş, I., Manea, M. & Gheorghe, M. InSAR surface deformation and numeric modeling unravel an active salt diapir in Southern Romania. *Sci. Rep.* **11**, 12091 (2021). 34103630 10.1038/s41598-021-91517-4 PMC8187355

[19] Frumkin, A. Uplift rate relative to base-levels of a salt diapir (Dead sea basin, Israel) as indicated by cave levels. *SP* **100**, 41–47 (1996).

[20] Frumkin, A. Formation and dating of a salt pillar in Mount Sedom diapir, Israel. *Geol Soc America Bull* preprint, 1 (2006).

[21] Pirazzoli, P. A. et al. Quaternary coral-reef terraces from Kish and Qeshm islands, Persian gulf: new radiometric ages and tectonic implications. *Quatern. Int.* **120**, 15–27 (2004).

[22] Bruthans, J. et al. Holocene marine terraces on two salt diapirs in the Persian gulf, iran: age, depositional history and uplift rates. *J. Quaternary Sci.* **21**, 843–857 (2006).

[23] Bruthans, J. et al. Evolution of salt diapir and karst morphology during the last glacial cycle: effects of sea-level oscillation, diapir and regional uplift, and erosion (Persian gulf, Iran). *Geomorphology* **121**, 291–304 (2010).

[24] Bruthans, J., Filippi, M., Slavík, M., Závada, P. & Zare, M. Rapid evolution of salt glacier caves on a mountain diapir in a semiarid climate. *Geomorphology* **448**, 109058 (2024).

[25] Báldi, K. et al. Discovery of the Badenian evaporites inside the Carpathian arc: implications for global climate change and paratethys salinity. *Geol. Carpath.* **68**, 193–206 (2017).

[26] Tămaş, D. M., Schléder, Z., Krézsek, C., Man, S. & Filipescu, S. Understanding salt in orogenic settings: the evolution of ideas in the Romanian Carpathians. *AAPG Bull.* **102**, 941–958 (2018).

[27] Schleder, Z., Tamas, D. M., Krezsek, C., Arnberger, K. & Tulucan, A. Salt tectonics in the Bend zone segment of the Carpathian fold and thrust belt, Romania. *Int. J. Earth Sci. (Geol Rundsch)*. **108**, 1595–1614 (2019).

[28] Sordoillet, D., Weller, O., Rouge, N., Buatier, M. & Sizun, J. P. Earliest salt working in the world: from excavation to microscopy at the prehistoric sites of Ţolici and Lunca (Romania). *J. Archaeol. Sci.* **89**, 46–55 (2018).

[29] Giurgiu, I. Cea Mai mare peșteră în Sare Din România. *Natura României*. **3**, 234 (2010).

[30] Ponta, G. M. L. Eastern subcarpathians bend: salt karst: meledic plateau and slănic Prahova. In *Cave and Karst Systems of Romania* (eds Ponta, G. M. L. & Onac, B. P.) 451–454 (Springer International Publishing, 2019). 10.1007/978-3-319-90747-5_51.

[31] Tămaș, D. M. et al. Low-Angle Shear Within the Exposed Mânzălești Diapir, Romania: Salt Decapitation in the Eastern Carpathians Fold‐and‐Thrust Belt. *Tectonics* 40, (2021).

[32] SEGHEDI, A., RĂDAN, S. & BRICEAG, A. Salt-related Geological and Cultural Heritage in Romania. (2021). 10.5281/ZENODO.5795093

[33] Krézsek, C. et al. Structure and petroleum systems of the Eastern carpathians, Romania. *Mar. Pet. Geol.* **151**, 106179 (2023).

[34] Hippolyte, J. C. & Sandulescu, M. Paleostress characterization of the Wallachian phase in its type area (southeastern carpathians, Romania). *Tectonophysics* **263**, 235–248 (1996).

[35] Merten, S., Matenco, L., Foeken, J. P. T., Stuart, F. M. & Andriessen, P. A. M. From nappe stacking to out-of‐sequence postcollisional deformations: cretaceous to quaternary exhumation history of the SE Carpathians assessed by low‐temperature thermochronology. *Tectonics* **29**, 2009TC002550 (2010).

[36] Constantin, D. M., Cîrciumaru, E. & Vătămanu, V. V. The land’s susceptibility, due to atmospheric precipitations, within the catchment area of Câlnău. *Present Environ. Sustainable Dev.* **8**, 45–57 (2014).

[37] Ramsey, C. B. Bayesian analysis of radiocarbon dates. *Radiocarbon* **51**, 337–360 (2009).

[38] Reimer, P. J. et al. The IntCal20 Northern hemisphere radiocarbon age calibration curve (0–55 cal kBP). *Radiocarbon* **62**, 725–757 (2020).

[39] Hajdas, I. et al. Radiocarbon dating. *Nat. Rev. Methods Primers*. **1**, 62 (2021).

[40] Costantini, M. et al. European Ground Motion Service (EGMS). in. *IEEE International Geoscience and Remote Sensing Symposium IGARSS* 3293–3296 (IEEE, Brussels, Belgium, 2021). (2021). 10.1109/IGARSS47720.2021.9553562

[41] European Environment Agency European ground motion service: basic 2019–2023 (vector), europe, yearly, oct. 2024. *Eur. Environ. Agency*. 10.2909/7EB207D6-0A62-4280-B1CA-F4AD1D9F91C3 (2024).

[42] Grujinschi, C. & Observaţiuni Asupra ‘discordanţei’ Din Ivirea de Sare de La Baia baciului (Slănic Prahova). *Buletinul Intitutului De Petrol. Gaze Si Geologie*. **18**, 33–38 (1971).

[43] Stoica, C. & Gherasie, I. *Sarea Și Sărurile De Potasiu Și Magneziu Din România* (Intreprinderea poligrafica Oltenia, Craiova, 1981).

[44] Talbot, C. J., Medvedev, S., Alavi, M., Shahrivar, H. & Heidari, E. Salt extrusion at Kuh-e-Jahani, Iran, from June 1994 to November 1997. *SP* 174, 93–110 (2000).

[45] Hajdas, I., Guidobaldi, G., Haghipour, N. & Wyss, K. Sample selection, characterization and choice of treatment for accurate radiocarbon Analysis—Insights from the Eth laboratory. *Radiocarbon* **66**, 1152–1165 (2024).

[46] Němec, M., Wacker, L. & Gäggeler, H. Optimization of the graphitization process at Age-1. *Radiocarbon* **52**, 1380–1393 (2010).

[47] Ramsperger, U. et al. Lea—a novel low energy accelerator for ^14^C dating. *Radiocarbon* **66**, 1280–1288 (2024).

[48] van der Plicht, J. & Hogg, A. A note on reporting radiocarbon. *Quat. Geochronol.* **1**, 237–240 (2006).

[49] Sava, T. B. et al. Status report on the sample Preparation laboratory for radiocarbon dating at the new Bucharest roams center. *Radiocarbon* **61**, 649–658 (2019).

[50] Wacker, L., Němec, M. & Bourquin, J. A revolutionary graphitisation system: fully automated, compact and simple. *Nucl. Instrum. Methods Phys. Res., Sect. B*. **268**, 931–934 (2010).

[51] Poncoş, V. et al. An integrated platform for Ground-Motion mapping, local to regional scale; examples from SE Europe. *Remote Sens.* **14**, 1046 (2022).

[52] Crameri, F. Scientific colour maps. *Zenodo*10.5281/ZENODO.1243862 (2023).

[53] Crameri, F., Shephard, G. E. & Heron, P. J. The misuse of colour in science communication. *Nat. Commun.* **11**, 5444 (2020). 33116149 10.1038/s41467-020-19160-7 PMC7595127

